# Evaluation of nutritional risk factors in hematopoietic stem cell transplantation-eligible patients

**DOI:** 10.31744/einstein_journal/2020AO5075

**Published:** 2020-04-13

**Authors:** Ana Carolina Cavalcante Viana, Ana Patrícia Nogueira Aguiar, Brena Custodio Rodrigues, Priscila da Silva Mendonça, Fernanda Maria Machado Maia

**Affiliations:** 1 Hospital Universitário Walter Cantídio Universidade Federal do Ceará FortalezaCE Brazil Hospital Universitário Walter Cantídio , Universidade Federal do Ceará , Fortaleza , CE , Brazil .; 2 Universidade Federal do Ceará FortalezaCE Brazil Universidade Federal do Ceará , Fortaleza , CE , Brazil .

**Keywords:** Stem cell transplantation, Hematopoietic stem cells, Oxidative stress, Nutritional status, Food consumption, Sarcopenia

## Abstract

**Objective:**

To evaluate the nutritional risk factors in patients eligible for hematopoietic stem cell transplantation.

**Methods:**

A cross-sectional, descriptive study conducted with patients recruited from an hematology outpatient clinic. Study variables included demographic and clinical data, patient-generated global subjective assessment findings, anthropometric indicators, food intake and oxidative stress levels. The level of significance was set at 5% (p<0.05).

**Results:**

The sample comprised 72 patients, mean age of 48.93 years (14.5%). Multiple myeloma was the most prevalent condition (51.4%) in this sample. Most patients (55.6%) were overweight according to body mass index and at risk of cardiovascular disease according to waist circumference, conicity index and percentage of body fat. Sarcopenia was associated with risk of cardiovascular disease, hip-to-waist ratio (p=0.021), muscle strength depletion (p<0.001), food intake (p=0.023), reduced functional capacity (p=0.048), self-reported well-nourished status; p=0.044) and inadequate vitamin B6 (p=0.022) and manganese (p=0.026) intake. Elevated oxidative stress, detected in 33.3% of patients in this sample, was not associated with sarcopenia.

**Conclusion:**

Most patients in this sample were overweight and sarcopenic. Lean mass depletion was associated with risk of cardiovascular disease, reduced muscle strength, food intake changes, reduced functional capacity, self-reported well-nourished status and inadequate intake of vitamin B6 and manganese, but not with oxidative stress.

## INTRODUCTION

Hematopoietic stem cell transplantation (HSCT) is a therapeutic modality for treatment of bone marrow diseases and some neoplasms. Around 65 thousand HSCTs are performed annually worldwide. ^( 1 )^ According to the Brazilian transplantation registry, ^( 2 )^ 3,091 allogeneic and autologous transplants (1,239 and 1,852 respectively) were performed in Brazil, in 2018.

Transplantation success rates and prognosis are associated with nutritional status and oxidative stress. Eutrophic patients seem to have a good prognosis regarding graft *versus* host disease (GVHD) and elevated oxidative stress levels appear to be related to longer time to engraftment. ^( 3 )^

Sarcopenia (lean mass depletion) is another nutritional condition potentially associated with morbidity risks, longer hospital stay and hospital-acquired complications in chronic disease patients. Sarcopenia has been associated with high risk of solid tumor relapse and higher risk of non-relapse mortality in oncologic patients submitted to HSCT. ^( 4 )^

Proper food intake may help control oxidative stress and maintain or improve nutritional status. ^( 5 )^ Therefore, nutritional aspects must be understood for efficient multidisciplinary treatment of hematologic patients eligible for HSCT.

## OBJECTIVE

To evaluate nutritional risk factors in hematopoietic stem cell transplantation-eligible patients.

## METHODS

A cross-sectional, descriptive study with patients eligible for autologous or allogeneic HSCT seen at the Hematology Outpatient Clinic of a reference hospital, located in the city of Fortaleza (State of Ceara, Brazil). The convenience sample comprised patients seeking the service from August 1 ^st^ to November 3 ^rd^ , 2018 (data collection period), who agreed to participate in the study, regardless of sex. The target population of the outpatient clinic includes primarily adults and elderly; therefore, only patients aged ≥18 years were included. Other inclusion criteria were hematologic conditions amenable to HSCT and no use of enteral and/or parenteral nutrition.

Nutritional assessment and blood withdrawal were carried out during pre-HSCT visits. Food intake assessment was based on 24-hour diet records compiled every other day, including one weekend day.

Study variables included demographic (sex and age), clinical (initial diagnosis and type of HSCT indicated) and biochemical (oxidative stress) data, nutritional status, body mass index (BMI), percentage body fat, patient-generated subjective global assessment (PG-SGA), sarcopenia, skeletal muscle function, cardiovascular disease (CVD) risk and food intake.

Body mass index was classified according to patient age. ^( 6 , 7 )^ Percentage body fat was estimated from summed triceps, biceps, subscapular and suprailiac skin folds. ^( 8 )^

Waist circumference (WC), ^( 9 )^ waist-to-hip ratio (WHR) and conicity index (CI) ^( 10 )^ were used as CVD risk indicators.

Skeletal muscle function assessment was based on maximal voluntary handgrip strength measured using a hydraulic hand dynamometer (Saehan ^®^ ). Participants with handgrip strength values below the 5 ^th^ percentile were defined as at risk of muscle mass depletion. ^( 11 )^

Sarcopenia assessment was based on cutoffs adjusted for sex, BMI and dynamometry findings. Participants with dynamometry readings below the baseline value were defined as sarcopenic, ^( 12 )^ ( Table 1 ). Patient-generated subjective global assessment (PG-SGA) adapted for the Brazilian population was also used. ^( 13 )^

**Table 1:** Baseline values for risk of sarcopenia according to body mass index and dynamometry findings in hematopoietic stem cell transplantation-eligible patients

BMI	Dynamometry cutoffs (kg)
Male	
<24	<29
24.1-26	<30
26.1-28	<30
>28	<32
Female	
<23	≤17
23.1-26	<17.3
26.1-29	<18
>29	<21

BMI: body mass index.

Oxidative stress was estimated from thiobarbituric acid reactive substances (TBARS) measurement at the experimental nutrition laboratory of the *Universidade Estadual do Ceará* (UECE). ^( 14 )^ Levels were determined according to the standard curve of malondialdehyde (MDA). Patients were stratified according to mean MDA values. Elevated oxidative stress was defined as MDA >4.27μM/L.

Food intake data were extracted from two 24-hour food records including one weekend day. Nutrition analysis was conducted using the software dietWin Professional Plus ^®^ .

Energy, macronutrient, micronutrient and fiber requirements were based on the Dietary Reference Intake criteria. ^( 15 )^

Mean protein and total energy intake were analyzed by direct comparison with pre- and post-HSCT intake recommendations (1.5g/kg/day and 30 to 35Kcal/kg/day, protein and caloric intake, respectively). ^( 16 )^

Only patients agreeing to participate were included in sample calculation. Statistical analysis was conducted using the - SPSS, version 19.0. Data were expressed as frequencies, percentages and means. Data normality was investigated using the Kolmogorov-Smirnov test. The homogeneity assumption was evaluated using the Levene test. Associations between categorical variables were investigated using the Pearson’s χ ^2^ or the Fisher’s exact test (categories with values <5). The level of significance was set at 5% (p<0.05).

This research project was conducted in compliance with guidelines for research involving human beings (resolution 466/12 of *Conselho Nacional de Saúde* ). ^( 17 )^ This project was approved by the Ethics Committee (opinion no. 2.771.145, CAAE: 84897218.7.0000.5045.

## RESULTS

The sample comprised 72 patients eligible for HSCT. Forty-one (56.9%) patients were male, mean age of 48.93 (±14.5) years. Multiple myeloma (MM) was the most prevalent (51.4%) condition in patients in this study ( Table 2 ).

**Table 2:** Distribution of hematopoietic stem cell transplantation-eligible patients according to demographic and clinical data, nutritional status and presence of sarcopenia

Demographic and clinical data	n (%)	Mean±standard deviation
Age, years		48.9±14.5
Sex		
Male	41 (56.9)	
Female	31 (43.1)	
Diagnosis		
MM	37 (51.4)	
HL	11 (15.3)	
AML	7 (9.7)	
NHL	6 (8.3)	
ALL	5 (6.9)	
Others	6 (8.4)	
Type of transplantation		
Autologous	58 (80.6)	
Allogeneic	14 (19.4)	
Nutritional status according to BMI		
Undernourished	5 (6.9)	
Eutrophic	27 (37.5)	
Overweight	40 (55.6)	
Sarcopenia		
Yes	45 (62.5)	
No	27 (37.5)	

MM: multiple myeloma; HL: Hodgkin lymphoma; AML: acute myeloid leukemia; NHL: non-Hodgkin lymphoma; ALL: acute lymphocytic leukemia; BMI: body mass index.

Most patients (40; 55.6%) were overweight ( Table 2 ) and at risk of CVD, as follows: 69.4% according to WC (91.6±11.4), 91.7% according to CI (1.2±0.0) and 73.6% according to percentage body fat (29.8±7.2).

As regards nutritional status, 35 (48.6%) of self-reported (PG-SGA) well-nourished individuals were sarcopenic (p=0.044).

More than half of participants consumed adequate amounts of carbohydrates (84.1%; mean, 236.7±104.9g), protein (96.8%; mean, 87.4±42.0g) and lipids (69.8%; mean, 45.2±23.9g). However, fiber (mean, 22.3±13.6g) and caloric intake (mean, 1,703±684.7Kcal) were inappropriate in 71.4% and 87.3% of cases, respectively.

With respect to antioxidant micronutrients, inadequate intake of vitamin E (mean, 1.7±1.5mg), vitamin A (mean, 461.1±883.6mcg) and selenium (mean, 71.6±75.8µg) was detected in 100%, 95.2% and 52.4% of patients, respectively. Adequate intake of vitamin C (mean, 305.6±838.6mg) and zinc (mean, 9.7±4.5mg) was confirmed in 55.6% and 60.3% of patients, respectively.

Sarcopenia was diagnosed in 62.5% of HSCT-eligible patients in this sample ( Table 2 ). Of these, 26 (36.1%) were at risk of CVD according to WHR (p=0.021). Sarcopenia was also associated with muscle strength depletion, reduced self-reported (PG-SGA) functional capacity and self-reported (PG-SGA) food intake changes (p<0.001, p=0.048 and p=0.023, respectively; Table 3 ).

**Table 3:** Association between presence of sarcopenia, anthropometric variables and patient-generated subjective global assessment in hematopoietic stem cell transplantation-eligible patients

Variables	Sarcopenia			p value
	Yes	No	Total	
WHR				
At risk of CVD	26 (36.1)	8 (11.1)	34 (47.2)	0.021 ^†^
No CVD risk	19 (26.4)	19 (26.4)	38 (52.8)	
Total	45 (62.5)	27 (37.5)	72 (100.0)	
Dynamometry				
Depletion	26 (36.1)	0.0 (0.0)	26 (36.1)	<0.001 ^‡^
No depletion	19 (26.4)	27 (37.5)	46 (63.9)	
Total	45 (62.5)	27 (37.5)	72 (100.0)	
PG-SGA, food intake				
No change	21 (29.2)	20 (27.8)	41 (56.9)	0.023 ^†^
Change	24 (33.3)	7 (9.7)	31 (43.1)	
Total	45 (62.5)	27 (37.5)	72 (100.0)	
PG-SGA, functional capacity				
Altered	35 (48.6)	15 (20.8)	50 (69.4)	0.048 ^§^
Normal	10 (13.9)	12 (16.7)	22 (30.6)	
Total	45 (62.5)	27 (37.5)	72 (100.0)	
PG-SGA, nutritional status				
Well nourished	35 (48.6)	26 (36.1)	61 (84.7)	0.044 ^‡^
Undernourished	10 (13.9)	1 (1.4)	11 (15.3)	
Total	45 (62.5)	27 (37.5)	72 (100.0)	

Results expressed as n (%).

^†^ Pearson’s χ ^2^ test; ^‡^ Fisher’s exact test; ^§^ Pearson’s R test.

WHR: waist-to-hip ratio; CVD: cardiovascular disease; PG-SGA: patient-generated subjective global assessment.

Higher prevalence of inadequate vitamin B6 (mean 0.99±0.67mg) and low manganese (2.4±1.4mg) intake was observed among sarcopenic individuals (57.1% and 34.9%, p=0.022 and p=0.026, respectively ( Table 4 ).

**Table 4:** Association between presence of sarcopenia, vitamin B6 and manganese intake in hematopoietic stem cell transplantation-eligible patients

Macro/micronutrient intake	Yes	No	p value
Carbohydrates			
Inadequate	6 (9.5)	4 (6.3)	1.000*
Adequate	34 (54)	19 (30.2)	
Protein			
Inadequate	2 (3.2)	0	0.529*
Adequate	38 (60.3)	23 (36.5)	
Lipids			
Inadequate	11 (17.5)	8 (12.7)	0.578*
Adequate	29 (46)	15 (23.8)	
Fiber			
<Adequate intake	28 (44.4)	17 (27.0)	0.781*
≥Adequate intake	12 (19.1)	6 (9.5)	
Vitamin C			
Inadequate	18 (28.6)	10 (15.9)	0.559*
Adequate	22 (34.9)	13 (20.6)	
Vitamin A			
<RDA	39 (61.9)	21 (33.3)	0.299*
≥RDA	1 (1.6)	2 (3.2)	
Selenium			
Inadequate	22 (34.9)	11 (17.5)	0.387*
Probably adequate	18 (28.6)	12 (19.0)	
Vitamin E			
Inadequate	40 (63.5)	23 (36.5)	NA
Adequate	0	0	
Zinc			
Inadequate	18 (28.6)	7 (11.1)	0.193*
Adequate	22 (34.9)	16 (25.4)	
Vitamin B6			
Inadequate	36 (57.1)	15 (23.8)	0.022*
Adequate	4 (6.3)	8 (12.7)	
Manganese			
<Adequate intake	22 (34.9)	6 (9.5)	0.026 ^†^
≥Adequate intake	18 (28.6)	17 (27.0)	

Results expressed as n (%).

* Fisher’s exact test; ^†^ χ ^2^ test.

RDA: Recommended Dietary Allowances; NA: not applicable.

Food intake data were obtained from 63 out of 72 participants.

Elevated oxidative stress was detected in 24 (33.3%) of participants. However, oxidative stress (MDA) was not significantly associated with remaining study variables.

## DISCUSSION

Undernourishment and excess weight may affect HSCT success rates and postoperative clinical status of eligible patients. Excess weight (defined by BMI), detected in 55.6% of patients in this sample and reported in 67.4% of patients overall, ^( 18 )^ may be associated to higher risks of GVHD and low survival rates following allogeneic HSCT due to high dose chemotherapy and GVHD prophylaxis. ^( 3 )^

Risk of CVD was another finding of this study. Mean WC (91.6±11.4cm; CVD risk) was in keeping with values reported elsewhere (mean WC, 96.8±6.9cm in HSCT-eligible patients). ^( 19 )^

Sarcopenia was the most prevalent nutritional condition in patients in this sample (62.5% overall and 48.6% of patients with self-reported changes in functional capacity). Similar findings have been reported in a different study (sarcopenia and significantly reduced functional capacity in 50.6% of patients scheduled for HSCT; p=0.022). ^( 20 )^ High rates of sarcopenia in these patients may reflect hematological disease malignancy and resulting cachexia and muscle wasting. ^( 21 )^

Sarcopenia may be determined by bioimpedance, portable dynamometry (as in this study) and gait speed assessment. These are thought to be highly efficient, reliable and feasible measurements for elderly and adult populations alike. ^( 12 )^

According to PG-SGA data, 48.6% of patients in this sample were well nourished, albeit sarcopenic. Limitations aside (questionnaire length, patient compliance, etc.), PG-SGA in thought to be an effective tool for nutritional status assessment in cancer and chronic disease patients. ^( 22 )^ However, this method was not able to detect lean mass depletion.

Sarcopenia may occur in patients with normal or high percentage body fat. Loss of muscle function combined with fat tissue deposit is defined as sarcopenic obesity. Obese sarcopenic individuals suffering from chronic diseases, such as cancer, are prone to longer hospital stay and postoperative infections. ^( 23 )^

In sarcopenic obesity, inflammation may trigger molecular changes, such as increase protein degradation, reduced protein synthesis, increased rates of myocyte apoptosis and mitochondrial dysfunction. ^( 24 )^

Sarcopenia is thought to be predictor of relapse in patients with solid tumors, regardless of age or pretreatment comorbidity. A 1.7-fold increase in risk of nonrelapse mortality has been reported in sarcopenic compared to non-sarcopenic patients suffering from hematological diseases. Sarcopenia has also been associated with longer hospital stay (p<0.001). ^( 4 )^

Therefore, sarcopenia seems to be a significant factor in nonrelapse mortality following HSCT and may assist in decision making in different phases of the transplantation process, including conditioning regimen intensity, nutritional optimization, enhanced support and resistance training, in an effort to mitigate HSCT-related complications. ^( 4 )^

Muscle wasting is often related to insufficient nutrient intake. Therefore, treatment strategies should include proper food intake combined with protein and amino acid supplementation. ^( 24 )^

Low micronutrient intake may also impact lean mass profile, given the association between low vitamin B6 (pyridoxine; 57.1%) and manganese (34.9%) intake and sarcopenia. These micronutrients may play an important role in protein synthesis. Pyridoxal phosphate, the active form of pyridoxine, stabilizes carbon bonds in alpha-amino groups and supports amino acid synthesis and degradation. Pyridoxine deficiency has been associated with weakness and peripheral neuropathy. Lack of manganese, a vital element for proper amino acid and protein metabolism, may lead to skeletal abnormalities. ^( 25 )^

Oxidative imbalances may also lead to muscle atrophy and muscle metabolism changes. Preventive effects of antioxidant therapy have been reported. ^( 26 )^

Elevated oxidative stress (MDA >4.27μM/L) was noted 33.3% of patients in this sample. Similar result was found in another study, in which pre-HSCT patients with MM and lymphoma had a significantly higher mean baseline MDA when compared to the Control Group (p<0.05). Oxidative DNA damage and longer time to engrafting were also reported in these patients. ^( 27 )^

Longer time to engrafting in response to oxidative stress is deleterious to patients, since the longer the hospital stay, the higher the risk of infections and other complications associated with individual morbidity and mortality. ^( 1 )^

Food intake in thought to be a significant factor in oxidative stress modulation. Malondialdehyde and isoprostane are specific biomarkers for lipid oxidation. Antioxidant vitamin and mineral supplementation has been shown to positively impact MDA and isoprostane levels. ^( 5 )^

Low vitamin A intake has been reported in studies with HSCT-eligible patients. ^( 28 )^ Inadequate intake of antioxidant micronutrients such as vitamin E, vitamin A and selenium was documented in 100.0%, 95.2% and 52.4% of patients in this sample respectively. Similar antioxidant intake profile has been observed in oncologic patients (inadequate intake of vitamin A, zinc and vitamin E in 68.6%, 63.2% and 60.3% of patients, respectively). ^( 29 )^

Inclusion of antioxidant nutrients in cancer treatment protocols may favor tumor growth control and enhance antineoplastic drug activity, allowing for lower dosing and less adverse effects with no negative impacts on therapeutic outcomes. ^( 30 )^

Therefore, these nutrients may be highly beneficial, particularly in the conditioning phase, in which patients receive high-dose chemotherapy for elimination of malignant cancer cells from the hematopoietic and immune systems prior to transplantation. ^( 1 )^

As regards potential limitations, selection of a single method to diagnose sarcopenia in this study precluded comparison with findings of studies using other methods.

## CONCLUSION

Most hematopoietic stem cell transplantation-eligible patients in this sample were overweight and sarcopenic and had low oxidative stress levels. Presence of sarcopenia in patients self-reporting good nutritional status suggests lean mass deficiency in this population. Sarcopenia was also associated with risk of cardiovascular disease, reduced functional capacity, food intake changes and inadequate vitamin B6 and manganese intake.

Food intake in this population was not enough to satisfy energy, fiber or antioxidant vitamin needs.

Nutritional status assessment through sarcopenia investigation prior to hematopoietic stem cell transplantation may be an efficient tool for dietary counseling aimed to improve outcomes and prognosis in hematopoietic stem cell transplantation-eligible patients.
