# Supplementary material for: Evaluation of nutritional risk factors in hematopoietic stem cell transplantation-eligible patients
Source: Einstein (Sao Paulo). 2020 Apr 13;18:eAO5075. doi: 10.31744/einstein_journal/2020AO5075 (PMC7155940; doi:10.31744/einstein_journal/2020AO5075)
Supplement: Translation

# Translation: Avaliação dos fatores de riscos nutricionais em pacientes pré-transplante de célula-tronco hematopoiética

**Authors:** Ana Carolina Cavalcante Viana, Ana Patrícia Nogueira Aguiar, Brena Custodio Rodrigues, Priscila da Silva Mendonça, Fernanda Maria Machado Maia

**Affiliations:** 1 Brasil Hospital Universitário Walter Cantídio, Universidade Federal do Ceará, Fortaleza, CE, Brasil .; 2 Brasil Universidade Federal do Ceará, Fortaleza, CE, Brasil .

**Keywords:** Transplante de células-tronco, Células-tronco hematopoéticas, Estresse oxidativo, Estado nutricional, Consumo alimentar, Sarcopenia

## Abstract

**Objetivo:**

Avaliar os fatores de riscos nutricionais em pacientes pré-transplante de célula-tronco hematopoiética.

**Métodos:**

Estudo transversal, descritivo, realizado com pacientes de um ambulatório de hematologia. As variáveis estudadas foram demográficas, dados clínicos, avaliação subjetiva global produzida pelo próprio paciente, indicadores antropométricos, ingestão alimentar e estresse oxidativo. Os dados foram considerados estatisticamente significativos quando p<0,05.

**Resultados:**

A amostra do estudo foi constituída por 72 pacientes, com média de idade de 48,93 (14,5%) anos e com mieloma múltiplo (51,4%) como a patologia mais prevalente. Conforme índice de massa corporal, 55,6% dos pacientes encontravam-se com excesso de peso. De acordo com a circunferência da cintura, índice de conicidade e percentual de gordura corporal, houve prevalência de risco para doença cardiovascular. A sarcopenia foi associada ao risco de doença cardiovascular pela relação cintura/quadril (p=0,021), depleção da força muscular (p<0,001), além da ingestão alimentar (p=0,023), da capacidade funcional reduzida (p=0,048) e do diagnóstico de “bem nutrido“ (p=0,044), conforme a avaliação subjetiva global, e com consumo inadequado de vitamina B6 (p=0,022) e de manganês (p=0,026). Dentre os avaliados, 33,3% apresentaram estresse oxidativo elevado sem associação com sarcopenia.

**Conclusão:**

Pacientes do pré-transplante se apresentam, em sua maioria, com excesso de peso, mas com sarcopenia, estando essa ausência de massa magra associada a risco de doença cardiovascular, depleção da força muscular, alteração da ingestão alimentar, redução da capacidade funcional, classificação de “bem nutrido”, segundo a avaliação subjetiva global e consumo inadequado de vitamina B6 e manganês, não estando associada a estresse oxidativo.

## INTRODUÇÃO

O transplante de células-tronco hematopoiéticas (TCTH) é uma modalidade terapêutica realizada para tratar doenças hematológicas relacionadas à medula óssea e a alguns tipos neoplasias. No mundo, cerca de 65 mil TCTH são realizados por ano^( 1 )^e, no Brasil, de acordo com o registro brasileiro de transplantes,^( 2 )^em 2018, foram realizados 3.091 transplantes, dos quais foram 1.239 alogênicos e 1.852 autólogos.

O prognóstico e o sucesso do transplante estão associados tanto com o estado nutricional quanto com o estresse oxidativo. Os pacientes eutróficos parecem apresentar bom prognóstico em relação ao risco da ocorrência de doença do enxerto contra o hospedeiro (DECH), e estresse oxidativo elevado parece estar relacionado ao maior tempo para “pega” do enxerto.^( 3 )^

A sarcopenia (depleção de massa magra) também é uma condição nutricional que vem sendo investigada por estar associada a risco de morbidade, maior tempo de internação e complicações hospitalares em pacientes com doenças crônicas. Em pacientes oncológicos, ela foi correlacionada ao risco elevado de recidiva de tumores sólidos e maior risco de mortalidade (não associado à recidiva da doença) em pacientes pós -TCTH.^( 4 )^

Tendo em vista que o consumo alimentar adequado pode controlar o estresse oxidativo e manter ou melhorar o estado nutricional,^( 5 )^o conhecimento de aspectos nutricionais se faz necessário para a eficácia da conduta terapêutica multidisciplinar em doentes hematológicos com indicação de TCTH.

## OBJETIVO

Avaliar os fatores de riscos nutricionais em pacientes pré-transplante de células-tronco hematopoiéticas.

## MÉTODOS

Trata-se de estudo transversal e descritivo, realizado com pacientes com indicação de TCTH autólogo ou alogênico do Ambulatório de Hematologia de um hospital de referência localizado na cidade de Fortaleza (CE). A amostra de conveniência foi constituída pelos pacientes que procuraram o referido serviço durante o período de coleta de dados, que decorreu de 1^o^de agosto de 2018 a 3 de novembro de 2018, e aceitaram participar do estudo. Foram incluídos na pesquisa pacientes de ambos os sexos, com idade ≥18 anos, tendo em vista que o público-alvo do ambulatório referido são adultos e idosos, que apresentavam doenças hematológicas com indicação de TCTH e não se encontravam em nutrição por sonda enteral e/ou parenteral.

A avaliação nutricional e a coleta de sangue foram realizadas na consulta ambulatorial pré-TCTH. A avaliação do consumo alimentar foi realizada por meio de dois recordatórios de 24 horas, aplicados em dias alternados, incluindo 1 dia de final de semana.

As variáveis estudadas foram dados demográficos (sexo e idade), clínicos (diagnóstico inicial, tipo de TCTH indicado), bioquímicos (estresse oxidativo), estado nutricional, índice de massa corporal (IMC), percentagem de gordura corpórea, avaliação subjetiva global produzida pelo próprio paciente (ASG-PPP), presença de sarcopenia, função do músculo esquelético, risco de doenças cardiovasculares (DCV) e ingestão alimentar.

O IMC foi classificado de acordo com a idade dos pacientes.^( 6 , 7 )^A percentagem de gordura corpórea foi obtida pela soma das dobras cutâneas tricipital, biciptal, subescapular e suprailíaca.^( 8 )^

Circunferência da cintura (CC),^( 9 )^relação cintura/quadril (RQC) e o índice de conicidade (IC)^( 10 )^foram utilizados como indicadores do risco de DCV.

Para estimar a função do músculo esquelético, realizou-se aferição da força máxima voluntária de preensão manual, por meio de um dinamômetro hidráulico de mão da marca Saehan^®^. Classificaram-se com risco de depleção os participantes que apresentaram valores abaixo do percentil 5.^( 11 )^

Avaliou-se a sarcopenia usando os pontos de corte, considerando sexo, IMC e dinanometria do avaliado. Receberam a classificação de sarcopenia aqueles com o resultado da dinamometria inferior ao valor de referência,^( 12 )^conforme a tabela 1 . Além deste, realizou-se também a ASG-PPP adaptada para o Brasil.^( 13 )^

**Tabela 1:** Valores de referência para risco de sarcopenia, conforme índice de massa corporal e dinamometria, de pacientes em pré-transplante de células-tronco hematopoiéticas

IMC	Pontos de corte da dinamometria (kg)
Sexo masculino	
<24	<29
24,1-26	<30
26,1-28	<30
>28	<32
Sexo feminino	
<23	≤17
23,1-26	<17.3
26,1-29	<18
>29	<21

IMC: índice de massa corporal.

O estresse oxidativo foi analisado por meio das substâncias reativas ao ácido tiobarbitúrico (TBARS - *thiobarbituric acid reactive substances* ) no laboratório de nutrição experimental da Universidade Estadual do Ceará (UECE),^( 14 )^e os resultados foram calculados de acordo com curva padrão feita com malondialdeído (MDA). Os pacientes foram estratificados pela média obtida na análise do MDA, sendo considerado com estresse oxidativo elevado aqueles com MDA >4,27μM/L.

Dois recordatórios alimentares de 24 horas, sendo um de final de semana, foram utilizados para coletar dados referentes à ingestão alimentar do paciente, para posterior análise dos nutrientes obtidos, por meio do software *dietWin Professional Plus* ^®^.

Para analisar a adequação nutricional de energia, macronutrientes, micronutrientes e fibras, utilizaram-se os critérios de análise baseados nas *Dietary Reference Intake* .^( 15 )^

As médias do consumo de proteína e da ingestão energética total foram avaliadas por comparação direta com a recomendação de ingestão adequada para pré e pós-TCTH, que é de 1,5g/kg/dia para proteína e de 30 a 35Kcal/kg/dia para consumo calórico, respectivamente.^( 16 )^

Compuseram o cálculo da amostra somente os pacientes que aceitaram participar do estudo. Para a análise estatística, utilizou-se o software (SPSS), versão 19.0. Os dados foram expressos em frequências, percentuais e médias. Para verificar a normalidade dos dados, utilizou-se o teste de Kolmogorov-Smirnov e, para homogeneidade, realizou-se o teste de Levene. Para verificar a associação entre variáveis categóricas, utilizou-se o teste χ^2^de Pearson. Quando as categorias apresentaram valores <5, utilizou-se o teste exato de Fisher. Consideraram-se os dados estatisticamente significantes para p<0,05.

A pesquisa foi realizada conforme as diretrizes sobre pesquisas envolvendo seres humanos, estabelecidas pela resolução 466/12 do Conselho Nacional de Saúde.^( 17 )^O presente projeto foi submetido e aprovado pelo Comitê de Ética, sob parecer 2.771.145, CAAE: 84897218.7.0000.5045.

## RESULTADOS

A população do presente estudo, conforme tabela 2 , constituiu-se de 72 pacientes com indicação de TCTH; 41 (56,9%) eram do sexo masculino, com média de idade de 48,93 (± 14,5) anos. A patologia mais prevalente nesses indivíduos foi mieloma múltiplo (MM) (51,4%).

**Tabela 2:** Distribuição dos pacientes do pré-transplante de células-tronco hematopoiéticas, segundo dados demográficos, clínicos, estado nutricional e presença de sarcopenia

Dados demográficos, clínicos	n (%)	Média ± desvio padrão
Idade, anos		48,9±14,5
Sexo		
Masculino	41 (56,9)	
Feminino	31 (43,1)	
Diagnóstico		
MM	37 (51,4)	
LH	11 (15,3)	
LMA	7 (9,7)	
LNH	6 (8,3)	
LLA	5 (6,9)	
Outros	6 (8,4)	
Tipo de transplante		
Autólogo	58 (80,6)	
Alogênico	14 (19,4)	
Estado nutricional pelo IMC		
Desnutrição	5 (6,9)	
Eutrofia	27 (37,5)	
Excesso de peso	40 (55,6)	
Sarcopenia		
Sim	45 (62,5)	
Não	27 (37,5)	

MM: mieloma múltiplo; LH: linfoma de Hodgkin; LMA: leucemia mieloide aguda; LNH: linfoma não Hodgkin; LLA: leucemia linfoide aguda; IMC: índice de massa corporal.

A maioria dos pacientes (40; 55,6%) encontrava-se com excesso de peso ( Tabela 2 ) e foi diagnosticada com risco de DCV: 69,4% dos pacientes pela CC (91,6±11,4), 91,7% conforme IC (1,2±0,0) e 73,6% de acordo com percentagem de gordura corpórea (29,8±7,2).

De acordo com o estado nutricional pela ASG-PPP, 35 (48,6%) dos indivíduos que foram classificados como bem nutridos apresentaram-se sarcopênicos (p=0,044).

Constatou-se, ainda, que mais da metade dos avaliados apresentou consumo alimentar adequado de carboidrato (84,1%; média: 236,7±104,9g), proteína (96,8%; média: 87,4±42,0g) e lipídio (69,8%; média: 45,2±23,9g). Porém, 71,4% apresentaram consumo inadequado de fibra (média de 22,3±13,6g) e 87,3% de calorias (média de 1.703±684,7Kcal).

Em relação à ingestão de micronutrientes antioxidantes, todos os avaliados (100%) apresentaram consumo inadequado de vitamina E (média de 1,7±1,5mg), 95,2% de vitamina A (média de 461,1±883,6mcg) e 52,4% de selênio (média de 71,6±75,8µg). Obtiveram consumo adequado de vitamina C 55,6% (média de 305,6±838,6mg) e 60,3% de zinco (média de 9,7±4,5mg), respectivamente.

A sarcopenia apresentou-se em 62,5% dos pacientes pré-TCTH avaliados, demonstrado na tabela 2 . Dentre os diagnosticados com sarcopenia, 26 (36,1%) apresentaram risco de DCV, conforme RCQ (p=0,021). Essa condição também associou-se com depleção da força muscular (p<0,001), capacidade funcional reduzida, conforme ASG-PPP (p=0,048), e ingestão alimentar alterada, conforme a ASG-PPP (p=0,023), de acordo com a tabela 3 .

**Tabela 3:** Associação entre presença de sarcopenia e variáveis antropométricas e avaliação subjetiva global produzida pelo próprio paciente no período de pré- transplante de células-tronco hematopoiéticas

Variáveis	Sarcopenia			Valor de p
	Sim	Não	Total	
RCQ				
Com risco DCV	26 (36,1)	8 (11,1)	34 (47,2)	0,021^†^
Sem risco DCV	19 (26,4)	19 (26,4)	38 (52,8)	
Total	45 (62,5)	27 (37,5)	72 (100,0)	
Dinamometria				
Com depleção	26 (36,1)	0,0 (0,0)	26 (36,1)	<0,001^‡^
Sem depleção	19 (26,4)	27 (37,5)	46 (63,9)	
Total	45 (62,5)	27 (37,5)	72 (100,0)	
ASG-PPP, ingestão alimentar				
Inalterada	21 (29,2)	20 (27,8)	41 (56,9)	0,023^†^
Alterada	24 (33,3)	7 (9,7)	31 (43,1)	
Total	45 (62,5)	27 (37,5)	72 (100,0)	
ASG-PPP, capacidade funcional				
Alterada	35 (48,6)	15 (20,8)	50 (69,4)	0,048^§^
Normal	10 (13,9)	12 (16,7)	22 (30,6)	
Total	45 (62,5)	27 (37,5)	72 (100,0)	
ASG-PPP, stado nutricional				
Bem nutrido	35 (48,6)	26 (36,1)	61 (84,7)	0,044^‡^
Desnutrido	10 (13,9)	1 (1,4)	11 (15,3)	
Total	45 (62,5)	27 (37,5)	72 (100,0)	

Resultados expressos por n (%).

^†^teste χ^2^de Pearson;^‡^teste exato de Fisher;^§^teste de Pearson R.

RCQ: relação cintura/quadril; DCV: doença cardiovascular; ASG-PPP: avaliação subjetiva global produzida pelo próprio paciente.

Foi observada, também, maior prevalência de indivíduos com sarcopenia apresentando ingestão inadequada de vitamina B6 (57,1%, p=0,022) e abaixo da ingestão adequada de manganês (34,9%; p=0,026) ( Tabela 4 ); média de ingestão de vitamina B6 de 0,99±0,67mg e de manganês de 2,4±1,4mg.

**Tabela 4:** Associação entre presença de sarcopenia e consumo de vitamina B6 e manganês de pacientes em pré-transplante de células-tronco hematopoiéticas

Adequação dos macro/micronutrientes	Sim	Não	Valor de p
Carboidratos			
Inadequada	6 (9,5)	4 (6,3)	1,000*
Adequada	34 (54)	19 (30,2)	
Proteína			
Inadequada	2 (3,2)	0	0,529*
Adequada	38 (60,3)	23 (36,5)	
Lipídeos			
Inadequada	11 (17,5)	8 (12,7)	0,578*
Adequada	29 (46)	15 (23,8)	
Fibras			
<Ingestão adequada	28 (44,4)	17 (27,0)	0,781*
≥Ingestão adequada	12 (19,1)	6 (9,5)	
Vitamina C			
Inadequada	18 (28,6)	10 (15,9)	0,559*
Adequada	22 (34,9)	13 (20,6)	
Vitamina A			
<RDA	39 (61,9)	21 (33,3)	0,299*
≥RDA	1 (1,6)	2 (3,2)	
Selênio			
Inadequada	22 (34,9)	11 (17,5)	0,387*
Provavelmente adequada	18 (28,6)	12 (19,0)	
Vitamina E			
Inadequada	40 (63,5)	23 (36,5)	NA
Adequada	0	0	
Zinco			
Inadequada	18 (28,6)	7 (11,1)	0,193*
Adequada	22 (34,9)	16 (25,4)	
Vitamina B6			
Inadequada	36 (57,1)	15 (23,8)	0,022*
Adequada	4 (6,3)	8 (12,7)	
Manganês			
<Ingestão adequada	22 (34,9)	6 (9,5)	0,026^†^
≥Ingestão adequada	18 (28,6)	17 (27,0)	

Resultados expressos por n (%).

* teste exato de Fisher;^†^teste χ^2^.

RDA: *Recommended Dietary Allowances* ; NA: não se aplica.

Dos 72 participantes, 63 apresentaram dados do consumo alimentar.

Na avaliação do estresse oxidativo, 24 (33,3%) dos avaliados apresentaram estresse oxidativo elevado. No entanto, não houve associação significativa entre estresse oxidativo (MDA) e as outras variáveis estudadas.

## DISCUSSÃO

No paciente com indicação de TCTH, tanto a desnutrição como o excesso de peso podem interferir no sucesso do transplante e no estado clínico do paciente após o procedimento. O excesso de peso, conforme o IMC, observado em 55,6% dos pacientes avaliados e registrado em 67,4% de pacientes da literatura,^( 18 )^pode estar associado a um maior risco de ocorrência de DECH e baixa sobrevida após o TCTH alogênico, devido à quimioterapia de alta dose e à profilaxia de DECH.^( 3 )^

Outro achado, neste estudo, foi o risco de DCV. A média da CC foi de 91,6±11,4cm (risco de DCV), corroborando o encontrado em outra pesquisa, que obteve média de 96,8±6,9cm em pacientes pré-TCTH.^( 19 )^

A sarcopenia foi condição nutricional também evidenciada nesses pacientes. Dentre os participantes do estudo, 62,5% apresentaram sarcopenia e 48,6% daqueles que declararam alteração da capacidade funcional pelo ASG-PPP também se encontrava sarcopênicos. Esse resultado corroborou outro estudo, no qual 50,6% daqueles que aguardavam o TCTH apresentaram-se com sarcopenia e capacidade funcional significativamente mais baixa (p=0,022).^( 20 )^Esta condição foi comum entre esses pacientes, pelo fato de a doença hematológica ser, muitas vezes, maligna, cursando com caquexia, caracterizada por depleção muscular.^( 21 )^

A presença de sarcopenia pode ser localizada por meio da bioimpedância, da dinamometria portátil (método aderido no presente estudo) e do teste da velocidade da marcha. Esses parâmetros são considerados de alta eficácia, confiáveis e viáveis, tanto para a população idosa quanto para adultos não idosos.^( 12 )^

Conforme a classificação nutricional pela ASG-PPP, 48,6% dos pacientes foram enquadrados em bem nutridos, mas sarcopênicos. Apesar de a ASG-PPP apresentar algumas limitações (extensão do questionário, necessidade de colaboração do paciente etc.), esse método que é considerado ferramenta eficaz para avaliar o estado nutricional de pacientes com câncer e doenças crônicas,^( 22 )^não tendo sido, porém, capaz identificar a deficiência de massa magra.

A sarcopenia pode estar presente no paciente com gordura corporal normal ou aumentada. A perda de função muscular associada ao acúmulo de tecido adiposo recebe o nome de obesidade sarcopênica. Indivíduos com essa perda muscular com doença crônica, como o câncer, estão sujeitos a maior tempo de internação hospitalar e maior risco de infecção pós-operatória.^( 23 )^

Nessa condição nutricional, a inflamação é o fator desencadeante das alterações moleculares, como aumento da degradação de proteínas, redução da síntese proteica, aumento da apoptose dos miócitos e redução da regeneração muscular, em conjunto com disfunção mitocondrial.^( 24 )^

A sarcopenia está sendo vista como preditor de recidiva em pacientes com tumores sólidos, independente da idade ou comorbidade pré-tratamento. Em pacientes com doenças hematológicas, a sarcopenia foi associada com risco de 1,7 vez à mortalidade não relacionada à recidiva da doença (MNR) quando comparada àqueles com massa muscular normal. Essa condição também associou-se com maior tempo de internação (p<0,001).^( 4 )^

Dessa forma, essa condição é um importante fator para MRN pós-TCTH e pode ser usada para a tomada de decisões nas fases do transplante, como intensidade de condicionamento, otimização alimentar, aumento dos serviços de suporte e treinamento de resistência, tendo em vista a redução do risco de complicações relacionadas ao pós-TCTH.^( 4 )^

Essa perda de massa muscular, em muitos casos, está relacionada à ingestão insuficiente de nutrientes. Assim, incluem-se, em seu tratamento, o consumo alimentar adequado e a suplementação de proteínas e aminoácidos.^( 24 )^

A baixa ingestão de micronutrientes parece afetar também o perfil de massa magra, pois a baixa ingestão de vitamina B6 (ou pirodoxina) (57,1%) e de manganês (34,9%) associou-se à sarcopenia. Isso pode estar relacionado às importantes funções desses nutrientes sobre a proteína: o piridoxal fosfato, forma funcional da piridoxina, por exemplo, tem ação nos grupos alfa-amino do aminoácido, estabilizando ligações de carbono, além de auxiliar na síntese e na quebra de aminoácidos. Sua deficiência pode causar fraqueza e neuropatias periféricas. O manganês, por sua vez, é essencial para o metabolismo adequado dos aminoácidos e proteínas, e sua privação levam a anormalidades esqueléticas.^( 25 )^

Além da ingestão reduzida de nutrientes, o desequilíbrio oxidativo pode ser fator desencadeante de atrofia e distúrbios no metabolismo muscular. Estudos têm demonstrado que terapia com antioxidantes parece contribuir para a prevenção dessa perda.^( 26 )^

Conforme a avaliação do estresse oxidativo, 33,3% dos pacientes apresentaram estresse oxidativo elevado (>4,27μM/L). Resultado semelhante foi encontrado em outro estudo, no qual pacientes pré-TCTH portadores de MM e linfoma apresentaram média de MDA basal significativamente altos, quando comparados com o Grupo Controle (p<0,05). Outro achado, nesses pacientes, foi a associação do desequilíbrio oxidativo ao dano no DNA e ao maior tempo de enxertia.^( 27 )^

Essa relação do estresse oxidativo, o maior tempo de enxertia torna-se desfavorável para o paciente, pois, quanto maior o tempo internação, maior o risco de infecções e outras complicações relacionadas à morbimortalidade do indivíduo.^( 1 )^

A ingestão nutricional apresenta-se como fator importante na modulação do estresse oxidativo. A suplementação de vitaminas e minerais antioxidantes tem conseguido demonstrar efeitos positivos sobre MDA e isoprostanos, os quais são biomarcadores específicos, relacionados à oxidação de lipídeos.^( 5 )^

O baixo consumo de vitamina A também foi verificado em outra pesquisa realizada com pacientes no pré-TCTH.^( 28 )^Além disso, foi observada ingestão inadequada de micronutrientes antioxidantes, como vitamina E, vitamina A e selênio, por 100,0%, 95,2%, 52,4%, respectivamente, dos avaliados do presente estudo. Esse perfil de ingestão inadequada de antioxidantes também foi verificado em 68,6%, 63,2% e 60,3% para vitamina A, zinco e vitamina E, respectivamente, em pacientes oncológicos.^( 29 )^

Os nutrientes antioxidantes, quando associados ao tratamento do câncer, parecem ter efeitos benéficos no controle do crescimento tumoral e intensifica a ação das drogas antineoplásicas, favorecendo à redução da dose a ser administrada e amenizando, dessa forma, os efeitos colaterais, sem haver prejuízo nos fins terapêuticos.^( 30 )^

Dessa forma, o efeito desses nutrientes ganha importância, principalmente na fase de condicionamento, na qual o paciente é submetido a altas doses de quimioteraterapia, para erradicação das células malignas e do sistema hematopoético e imunológico, antes do transplante.^( 1 )^

Acerca das limitações encontradas nesse estudo, cita-se o uso de apenas um método de diagnóstico de sarcopenia, impossibilitando a associação com os outros métodos.

## CONCLUSÃO

Os pacientes no pré-transplante de células-tronco hematopoiéticas apresentaram-se, em sua maioria, com excesso de peso, baixo estresse oxidativo e sarcopenia, a qual encontrou-se associada com o estado nutricional de bem nutridos pela avaliação subjetiva global produzida pelo próprio paciente, apontando deficiência de massa magra nessa população. Além disso, essa alteração nutricional está associada a risco de doenças cardiovasculares, redução da capacidade funcional, alteração da ingestão alimentar e ingestão inadequada de vitamina B6 e manganês.

O consumo alimentar nessa população encontrou-se inadequado em relação às necessidades calóricas, fibras e vitaminas antioxidantes.

Dessa forma, a avaliação nutricional do paciente na fase pré-transplante de células tronco hematopoiéticas, por meio da presença de sarcopenia, parece ser boa ferramenta para o aconselhamento dietético, visando à melhora e ao prognóstico desses pacientes.
